# Comparing the effect of tissue adhesive and suturing material on collagen I/III ratio in abdominal skin wounds: an experimental study

**DOI:** 10.1097/MS9.0000000000001206

**Published:** 2023-09-20

**Authors:** Imam Sofii, Aditya Rifqi Fauzi

**Affiliations:** a Division of Digestive Surgery, Department of Surgery, Faculty of Medicine, Public Health and Nursing, Universitas Gadjah Mada/Dr. Sardjito Hospital, Yogyakarta; b Department of Surgery, Faculty of Medicine, University of Indonesia/Dr. Cipto Mangunkusumo Hospital, Jakarta 104401, Indonesia

**Keywords:** Abdominal skin closure, collagen I/III expression ratio, suturing material, tissue adhesive, wound dehiscence

## Abstract

**Background:**

The skin closure procedure should be technically simple, acceptable, quick, and cost-effective. Sutures remain the technique’s mainstay, however tissue adhesive is becoming more used in clinical practice. Collagen ratios of types I and III play a significant role as postoperative wound healing parameters. Here, the authors aim to examine the collagen I/III ratio of tissue adhesive vs. non-absorbable sutures for abdominal skin closure in Wistar albino rats.

**Material and methods:**

The authors allocated 20 rats into four experimental groups. Wounds in groups 1 and 3 were sealed with tissue adhesive (cyanoacrylate), while those in groups 2 and 4 were closed using suture material (monofilament non-absorbable nylon). Groups 1 and 2 were sacrificed on postoperative day (POD) 4, while those in groups 3 and 4 were euthanized on POD 7. Skin samples (1×0.5 cm) were collected for analysis, and the collagen I/III ratios were determined using immunohistochemistry staining techniques.

**Results:**

The levels of collagen I and III expression did not exhibit statistically significant differences between tissue adhesive and nylon suture groups at either POD 4 (*P*=0.052, *P*=0.513) or POD 7 (*P*=0.125, *P*=0.80). Similarly, the collagen I/III ratio did not significantly differ between the two groups at POD 4 (1.23±2.26 vs. 0.70±0.24; *P*=0.47) or POD 7 (0.68±0.96 vs. 0.77±1.22; *P*=0.857).

**Conclusions:**

There were no statistical significance difference in collagen I/III ratio between the tissue adhesive and suture material groups, suggesting that the choice of wound closure material may not influence the abdominal skin closure.

## Introduction

Highlights

- Collagen I/III ratio in skin closure using tissue adhesive vs. non-absorbable sutures.
- No significant differences found in collagen I/III ratios between tissue adhesive and suture groups.
- Suggests both methods may be equally effective for abdominal skin closure.

Adequate wound closure is essential for minimizing wound complications and dehiscence. The incision site is a crucial aspect of surgical procedures, and proper closure and management of the surgical area are vital determinants of successful wound healing and surgical outcome. Traditional suture techniques involve the application of stress during needle penetration and create a “wick” through which bacteria can potentially access underlying tissues, thereby increasing the risk of infection. Moreover, the presence of suture material itself can lead to complications such as stitch abscess, epithelial inclusion cysts, and undesirable scarring. It is also worth noting that suture techniques involve the invasion of the underlying epithelial layer, which can further contribute to these adverse effects^1^.

There has been significant progress in the field of wound closure techniques over the years. In addition to traditional suture methods, various alternatives such as staples, tapes, and adhesive compounds have been developed. The ideal skin closure method should be straightforward, safe, efficient, cost-effective, painless, bactericidal, and produce aesthetically pleasing scarring^2^.

Postoperative surgical site infection, obesity, and an abdominal aortic aneurysm are all known risk factors for the development of skin dehiscence. However, the material used to close an abdominal wall incision has been identified as a particularly significant factor^3–5^.

Dysregulation of collagen synthesis can impair the skin wound closure process. A decreased collagen I/III ratio has been linked to the formation of unstable scars^6^.

Collagen is a vital component of the extracellular matrix that helps to preserve tissue elasticity and tensile strength. Collagen type I is a robust form of collagen that is widely distributed in the human body, including in the fascia, skin, ligaments, and fibrous tissue, and is responsible for the mechanical strength of these tissues. Collagen type III, on the other hand, is present in smaller amounts at the beginning of wound healing and is weaker in terms of mechanical properties. During the healing process, collagen type III in the wound is replaced by stronger collagen type I. Therefore, the ratios of collagen types I and III are crucial indicators of postoperative wound healing^7^. The primary aim of this study was to compare the collagen type I/III ratio between tissue adhesive and suture material for abdominal skin closure in Wistar albino rats.

## Material and methods

### Animal subjects

Twenty male *Rattus novergicus* rats, weighing 170–200 g and aged 2–3 months, were kept under normal conditions and isolated for seven days. Rats that were unhealthy, developed infections, or died during the study were excluded from the analysis. No rat was excluded due to health issues or death during the seven-day isolation period prior to the experimental procedure. Twenty rats were randomly assigned to one of the four experimental groups using a simple random sampling method.

### Experimental procedure

All samples were obtained and incised at the Anatomy Department of our institution. The methods used in this study were based on those employed in a previous study conducted by our group^8^. The rats were sedated with an intramuscular injection of 60 mg/kg after aseptic-antiseptic action with 1% povidone iodine prior to the incision of the wound. The depth of anaesthesia was assessed by monitoring the pedal withdrawal reflex, and additional anaesthesia was administered as needed to maintain adequate sedation during the procedure. The incision was made using a surgical blade, and bleeding was controlled using sterile gauze and pressure. Care was taken to minimize any discomfort or distress experienced by the animals during the procedure, and analgesics were administered postoperatively to manage pain and promote recovery.

Prior to skin sample collection, the hair on the abdominal area of each rat was shaved using an electric clipper. All skin samples were incised in the shape of a rectangle (6 ×3 cm) using a surgical blade size 10. A simple random sampling method was used to divide each sample into four groups. The tissue adhesive (cyanoacrylate/Histoacryl) was applied using a single-use applicator, and ~0.1 ml of adhesive was used for each rat. Group 1 rats had their wounds repaired with tissue adhesive (cyanoacrylate/Histoacryl) and were sacrificed on postoperative day (POD) 4. Group 2 rats were suture closed with suture material (4-0 non-absorbable monofilament nylon) and were sacrificed on POD 4. Group 3 rats had their wounds repaired with tissue adhesive (cyanoacrylate/Histoacryl) and were sacrificed on POD 7. Group 4 rats were suture closed with suture material (non-absorbable monofilament nylon) and were sacrificed on POD 7. Following euthanasia, skin samples (1×0.5 cm) were collected and immunohistochemically stained for collagen I and III.

Collagen expression was measured using immunohistochemistry staining. Briefly, skin samples were fixed in 4% paraformaldehyde for 24 h, embedded in paraffin, and cut into 4-μm-thick sections. After deparaffinization and rehydration, the sections were treated with 3% hydrogen peroxide to block endogenous peroxidase activity, and then incubated with primary antibodies against collagen I (Abcam, ab34710) and collagen III (Abcam, ab7778) at a dilution of 1:200 at 4°C overnight. The sections were then incubated with a secondary antibody, followed by incubation with avidin-biotin-peroxidase complex reagent. Finally, the sections were developed with 3,3’-diaminobenzidine and counterstained with hematoxylin. Images were captured using an Olympus microscope, and the collagen I/III ratio was quantified using an Adobe Reader counter. Multiple locations were not sampled in order to allow for comparison. The amounts of collagen I and III were then quantified using an Adobe Reader counter. Multiple locations were not sampled in order to allow for comparison. This study is fully compliant with the ARRIVE criteria^9^.

### Statistical analysis

Independent *t*-tests were conducted to examine the relationship between tissue adhesive and suture material and collagen I/III ratios. In this study, a *P* value of less than 0.05 was considered statistically significant, and a 95% CI was used for all analyses.

## Results

Collagen I expression in rat skin wounds closed with tissue adhesive and suture material at POD 4 was almost reached statistically significant level, 57.30±20.92 and 42.87±6.57, respectively (*P*=0.052), while collagen I expression in rat skin wounds closed with tissue adhesive and suture material at POD 7 was 45.47±17.26 and 49.63±9.48, respectively (*P*=0.513) (Table 1). Additionally, collagen III expression did not differ significantly between rats with wounds closed with tissue adhesive and suture material at either POD 4 (46.53±9.24 vs. 61.13±27.15; *P*=0.125) or POD 7 (66.40±18.07 vs. 64.80±7.79; *P*=0.800) (Table 1).

**Table 1:** Comparison of collagen I and III expressions between tissue adhesive and suture material on PODs 4 and 7

	Collagen I expressions			Collagen III expressions		
	Tissue adhesive (mean±SD)	Suturing material (mean±SD)	*P*	Tissue adhesive (mean±SD)	Suturing material (mean±SD)	*P*
Day 4	57.30±20.92	42.87±6.57	0.052	46.53±9.24	61.13±27.15	0.125
Day 7	45.47±17.26	49.63±9.48	0.513	66.40±18.07	64.80±7.79	0.800

POD, postoperative day.

*P*<0.05 is considered significant.

Additionally, the collagen I/III ratio did not differ significantly between the two groups at either POD 4 (1.23±2.26 vs. 0.70±0.24; *P*=0.470) or POD 7 (0.68±0.96 vs. 0.77±1.22; *P*=0.800) (Table 2).

**Table 2:** Comparison of the collagen I/III ratio between tissue adhesive and suture material on PODs 4 and 7

	Collagen I/III ratio		
	Tissue adhesive (mean±SD)	Suturing material (mean±SD)	*P*
Day 4	1.23±2.26	0.70±0.24	0.470
Day 7	0.68±0.96	0.77±1.22	0.857

POD, postoperative day.

*P*<0.05 is considered significant.

## Discussion

In this study, we demonstrate that tissue adhesive and nylon did not differ significantly on the effects on the healing of abdominal skin wounds, as evidenced by comparable collagen I/III expression ratios. Collagen metabolism plays a crucial role in the repair of the abdominal wall, and maturation and degradation of collagen are particularly important processes. A decreased collagen I/III ratio indicates a deficiency in mature collagen, and increased activity of matrix metalloproteinases, which are responsible for collagen type I denaturation, has been implicated in the development of abdominal hernias^10^.

Villagomez *et al*.^11^ conducted a similar experimental study in rats that compared cyanoacrylate and traditional poliglecaprone sutures in surgical wound closure, and found no significant difference between the two groups, indicating that both methods may be effective for treating surgical wounds. The process of healing involves several steps, including haemostasis, inflammation, proliferation, and remodelling^12^. Sutures or adhesives are used to accurately approximate incisional wound margins in order to reduce strain and facilitate healing^13^.

From an aesthetic standpoint, a notable advantage of topical adhesives like cyanoacrylate lies in the inconspicuous absence of track marks, particularly when considering the realms of facial aesthetics and paediatrics. For the patient, the remarkable ability to shower soon after the operation without any apprehension regarding incision compromise bestows remarkable benefits^14^.

The limitations of cyanoacrylate were primarily of a technical nature, demanding cautious attention to mitigate their impact. According to the existing literature, this adhesive has been observed to permeate the wound edges, hindering the healing process and influencing scar appearance due to foreign body reactions^15^. Remarkably, Asai *et al*. noted that ~1.6% of patients exhibited allergic contact dermatitis following the initial application of cyanoacrylate tissue adhesive^16^. Moreover, Bitterman and Sandhu documented a papulovesicular rash at the application site, arising 2 weeks postoperatively, which was attributed to residual glue remaining at the incision site; fortunately, the condition showed improvement upon thorough washing of the adhesive^17^.

Collagen type III, like collagen type I, forms fibrils and is produced by fibroblasts. However, collagen type III has a less rigid structure and lower strength than collagen type I^18^. Collagen type III levels increase during the early stages of wound healing, particularly during the haemostasis and early inflammatory phases. In contrast, collagen type I, which has a stronger structure and greater strength, gradually increases over the same time period, replacing collagen type III^7^. Therefore, disruptions in collagen metabolism can impair skin wound healing and increase the risk of skin dehiscence^19^. Delayed reduction of collagen III and modest increases in collagen I may indicate delayed wound healing

Suture closure is a safe and effective method for wound closure, but it can be time-consuming and dependent on the skill of the operator, with the risk of needle stick injury. In addition, suture removal requires an additional procedure, and the timing of suture removal can affect the degree of scarring^20^. Early suture removal may result in poor wound tensile strength and a wider scar, while late removal may cause scar formation due to inflammation^21^. n-butyl-2-cyanoacrylate is a tissue adhesive that has been used as an alternative to sutures for over 40 years. In liquid form, n-butyl-2-cyanoacrylate is monomeric, but upon application to tissue, it polymerizes and forms a strong bond between the wound edges^22,23^. Tissue adhesive provides a waterproof and antibacterial barrier that is easier and faster to apply than suture closure. It does not need to be removed since the wound naturally sloughs off following re-epithelialization. Some research suggests that it may also improve aesthetic outcomes^13^.

In a randomized trial conducted by Tandon *et al*.^24^, it was found that there were no statistically significant differences in the evaluated outcomes between wounds that were closed solely with sutures and those that received a combination of sutures and adjunctive tissue adhesive in the context of paediatric surgical wounds. However, in a study conducted by Roguljic and colleagues, it was revealed that cyanoacrylate-based adhesion mixtures may offer potential advantages over conventional skin suturing methods in the immediate postoperative period concerning the aesthetic outcome of surgical wound closure following open carpal tunnel syndrome decompression. Results indicated that at both 2 and 6 weeks postoperatively, the cyanoacrylate-based adhesion mixtures displayed superior cosmetic appearance^25^.

There are several limitations to our study. Skin dehiscence can be caused by various factors, including wound tension, increased abdominal pressure, malnutrition, and suture material. In addition, the animal model used in this study is not a skin dehiscence model, which limits the generalizability of our findings to the clinical treatment of skin dehiscence. Therefore, there is a significant gap between our results and their potential therapeutic use.

## Conclusions

There were no statistical significance difference in collagen I/III ratio between the tissue adhesive and suture material groups, suggesting that the choice of wound closure material may not influence the abdominal skin closure.

## Limitations

Our study used a relatively small number of rats and only 1-week observation.

## Ethical approval

The study protocol was approved by the Medical and Health Research Ethics Committee of our institution (KE/FK/0151/EC/2018).

## Consent

Not applicable.

## Sources of funding

The authors declare that this study had no funding source.

## Author contribution

I.S. conceived the study and critically revised the manuscript for important intellectual content. A.R.F. drafted the manuscript. All authors read and approved the final draft. All authors facilitated all project-related tasks.

## Conflicts of interest disclosure

No potential conflict of interest relevant to this article was reported.

## Research registration unique identifying number (UIN)

Not applicable.

## Guarantor

Imam Sofii.

## Data availability statement

Study data are available upon reasonable request.

## Provenance and peer review

Not commissioned, externally peer-reviewed.
